# To broaden the horizon of the Journal of Educational Evaluation for Health Professions

**DOI:** 10.3352/jeehp.2006.3.1

**Published:** 2006-07-07

**Authors:** Sun Huh

**Affiliations:** Department of Parasitology, College of Medicine and Institute of Medical Educations, Hallym University, Chuncheon, Korea.

Dear Readers and Researchers,

I am very happy to announce the adoption of a new format for the Journal of Educational Evaluation for Health Professions, which comprises three major changes. First, the language has been changed from Korean to English. Second, it will be published only on the World Wide Web. Third, the PubMed-Central XML format has been adopted, which is the standard XML for open-access medical journals. The criteria for acceptable material are detailed in the aims and scope section. I will encourage the publication of papers on the application of psychometric tools and nationwide data analysis. In addition to research articles, the journal will also publish case reports, brief reports, reviews, opinions, technical reports, software, letters to the editor, and book reviews, which will be of practical use to teachers. This change is a consequence of an information-hungry world that seeks easy and rapid access to practical and useful information.

I hope to soon publish review articles on the status and development of the national examinations for health professionals in several countries, since the international exchange of health professionals and mutual license accreditation is a hot topic according to the Doha Development Agenda (DDA).

In addition, I would like to make the journal an interesting and pleasant experience. I welcome articles from all over the world. The aim is to make the journal an effective forum for information exchange among health professionals.

Best wishes.

